# Mobile Robot Localization Based on the PSO Algorithm with Local Minima Avoiding the Fitness Function

**DOI:** 10.3390/s25206283

**Published:** 2025-10-10

**Authors:** Božidar Bratina, Dušan Fister, Suzana Uran, Izidor Mlakar, Erik Rot Weiss, Kristijan Korez, Riko Šafarič

**Affiliations:** 1 Faculty of Electrical Engineering and Computer Science, University of Maribor, Koroška cesta 46, SI-2000 Maribor, Slovenia; bozidar.bratina@um.si (B.B.); suzana.uran@um.si (S.U.); izidor.mlakar@um.si (I.M.); riko.safaric@um.si (R.Š.); 2 HSE Invest, d.o.o., Obrežna ulica 170, SI-2000 Maribor, Slovenia; erik.rot-weiss@hse-invest.si; 3 Piktronik d.o.o., Cesta k Tamu 17, SI-2000 Maribor, Slovenia; korez@piktronik.com

**Keywords:** mobile robot localization, PSO algorithm, avoiding the global minima

## Abstract

Localization of a semi-humanoid mobile robot Pepper is proposed based on the particle swarm optimization algorithm (PSO) that is robust to the disturbance perturbations of LIDAR-measured distances from the mobile robot to the walls of the robot real laboratory workspace. The novel PSO, with the avoiding local minima algorithm (PSO-ALM), uses a novel fitness function that can prevent the PSO search from trapping into the local minima and thus prevent the mobile robot from misidentifying the actual location. The fitness function penalizes nonsense solutions by introducing continuous integrity checks of solutions between two different consecutive locations. The proposed methodology enables accurate and real-time global localization of a mobile robot, given the underlying a priori map, with a consistent and predictable time complexity. Numerical simulations and real-world laboratory experiments with different a priori map accuracies have been conducted to prove the proper functioning of the method. The results have been compared with the benchmarks, i.e., the plain vanilla PSO and the built-in robot’s odometrical method, a genetic algorithm with included elitism and adaptive mutation rate (GA), the same GA algorithm with the included ALM algorithm (GA-ALM), the state-of-the-art plain vanilla golden eagle optimization (GEO) algorithm, and the same GEO algorithm with the added ALM algorithm (GEO-ALM). The results showed similar performance with the odometrical method right after recalibration and significantly better performance after some traveled distance. The GA and GEO algorithms with or without the ALM extension gave us similar results according to the accuracy of localization. The optimization algorithms’ performance with added ALM algorithms was much better at not getting caught in the local minimum, while the PSO-ALM algorithm gave us the overall best results.

## 1. Introduction

Localization and navigation in the workspace environment are natural processes for people and other species. Robots, however, must use a sample of sensors to be able to locate the workspace automatically, build an image of the workspace, and place themselves within this workspace. Simultaneous localization and mapping (SLAM) is a process of detecting either the unknown or known workspace and placing themself into it. Many sensors exist for conducting SLAM, mainly with cameras (monocular, stereo, and RGB-D) and time-of-flight sensors (light detection and ranging (LIDAR), radar, ultrasound, laser, etc.). The localization methods of today’s mobile robots can be divided into those that utilize internal or mounted sensors like LIDARs, inertial measurement units (IMUs), encoders, and cameras and those that rely on external radio frequency sensors such as Wi-Fi, Bluetooth, Ultra-Wideband, etc. [1,2]. For many localization cases, the limitation to traditional methods is predetermined by hardware, using on-board sensors that face difficulties in a real-time environment. Recent advances in hardware and computing methods have enabled many artificial intelligence methods that can be used for a robot’s localization. A review paper by Wang and Ahmad [3] divided these into Deep Learning (GAN, CNN, and RNN), Machine Learning (Reinforcement Learning and SVM), and Evolutionary and Swarm Intelligence methods (such as PSO, GA, DE, etc.). Most papers that have presented AI-based localization solutions operated with visual and odometrical information for mapping and/or localization, whereas our work relies on LIDARs measurements and evolutionary/swarm algorithms to find the robot’s position and orientation. In such a case a map of the mobile robot environment (workspace) must be known or has to be obtained by other mapping methods.

Once the mapping is performed and a so-called a priori map is built, the localization remains. Localization in a low-signal-to-noise ratio workspace is a misleading task, as the robot may not recognize several similar potential locations on the map but, instead, select one of them sporadically, not knowing that the location is incorrect. A simple remediation to this is to follow the path continuously and check the long-term integrity of a solution constantly so that, eventually, only a single solution exists.

The challenge we are addressing is a reliable and real-time localization of a semi-humanoid robot that is equipped with three independent LIDAR sensors, given the underlying a priori map. We have realized that the classical odometry integrates severe offset with respect to the distance traveled, which demands frequent recalibrations.

Moreno et al. [4] mentioned that, for evolutionary optimization algorithms, a fitness function based on the mean square error function does not perform well for mobile robot localization due to the following:

- Sensor accuracy and the number of sensors limit the possibility of discriminating between several fitness function local minima and a global minimum.
- The geometrical similarities of the robot workspace produce high numbers of possible mobile robot pose solutions.

We will show in our paper that one more reason exists that an optimization algorithm (OA) cannot discriminate between local minima and a global minimum if the mapping of the robot workspace is not measured accurately enough.

A lot of papers, in spite of their authors recognizing the problems mentioned by Moreno et al. [4], have not found solutions to discriminating between the local minima and global minimum [5,6,7], so their solutions were limited in practice. Zhang et al. [5] tried to address this problem by introducing a hybrid algorithm based on two evolutionary algorithms (the firefly algorithm and a genetic algorithm) but only presented the simulation results. Zhang et al. [8] found a solution for the discrimination of local and global minima, but his fitness function was complicated, so the average time to find the global minimum was 2.41 s in the Matlab environment, while our algorithm was executed a few times faster (0.5 s in the Python 2.7.18 environment), while the position and orientation errors were almost the same. When the geometrical similarities of the robot workspace produced a higher number of possible mobile robot pose solutions, Zhang et al.’s [8] algorithm needed to investigate from several nearby positions to find the global minimum, while our algorithm needed only two positions and, therefore, decreased the overall elapsed time to a maximum of 1 s in the Python environment.

The following literature review involved research on mobile robot localization with a known a priori map range. Havangi [6] implemented a practical case study of the mobile robot localization between the particle filter (PF) and extended Kalman filter (EKF) as benchmarks and PSO-driven robot localization. The PSO-based localization proved superior in terms of accuracy and consistency. Zhang et al. [8] presented an improved PF based on the PSO for mobile robot localization for an indoor Intel Research Lab public dataset and Fort AP Hill [9]. Chung et al. [10] implemented an improved Adaptive Monte Carlo Localization (AMCL) for tracking and localizing the robot’s position in two dimensions that is equipped with laser sensors and range finders. Ji et al. [11] employed pose tracking and localization based on a camera plane extraction in the camera coordinates and comparing these to a real-world map. De Sá et al. [12] showed an example of range-based mobile robot localization within a wireless network of stationary anchors with known positions. Using basic triangulation, the mobile robot position can be determined by using the min–max method and the PSO. Worley [13] implemented autonomous localization for water pipe inspection mobile robots. These robots need to define the absolute location based on several properties, such as pipe length, radius, interconnections, and others, and compare these to a known a priori map. A pose graph optimization method with fusion of acoustic information was used by the authors for efficient localization.

The work by Vahdat et al. [14] stipulated the importance of global (absolute) robot localization. The authors used three algorithms, Monte Carlo localization (MCL), differential evolution (DE), and particle swarm optimization (PSO). In a given case study, the DE proved to be most successful between the three. The authors reported that the DE underwent faster convergence than the PSO, as well as scored a higher convergence rate. Begum et al. [15] introduced the fuzzy evolutionary SLAM algorithm. A genetic algorithm was utilized as an OA for searching for the optimal robot pose, while fuzzy logic was utilized to handle the uncertainties in the robot’s pose. The robot was equipped with laser range finders, similar to a LIDAR, of up to 10 m of effective distance. The authors proposed that new position updates should be conducted for each 0.5–1 m of travel or a 20 to 40 degree change in rotation. Again, the authors emphasized the importance of solving the robot’s pose problem as a global optimization problem. Bian et al. [16] showed an example of a multi-objective PSO for FastSLAM, called FastSLAM-MO-PSO, especially useful for high noise and large-scale maps. Carvalho et al. [17] introduced the perfect match global localization (PMGL) metric for evaluation of the fitness of several hypothetical robot poses displaced over the map. The authors argued that the efficacy of the PMGL may vary across several scenarios. For a sample of maps, the PSO performed similarly to the GA. However, for one of the maps, the PSO performed significantly worse than the GA. The authors therefore highlighted the GA as the most intuitive solution for global localization. In the last five years, novel state-of-the-art swarm intelligence algorithms, such as golden eagle optimizer (GEO) [18], were proposed, with their performance validated in the robotics field [19].

The novelties of this paper are as follow:

- We upgraded the PSO algorithm successfully with an innovative search strategy that can discriminate between the local minima and the global minimum.
- A localization algorithm based on the PSO algorithm with an algorithm for avoiding the local minima was tested in the real laboratory workspace, and the results show that it outperformed the odometrical algorithm and OA’s benchmarks.
- The average overall elapsed time to calculate a new position was between $t_{e}=0.5$ and $t_{e}=1$ s in the Python environment.

This paper is structured as follows: the Section 2 presents the fundamentals of the Pepper robot, its LIDAR sensors and cameras, and the OAs, i.e., the PSO and two benchmark OAs. The Section 3 present the organization of the practical laboratory tests. Then, a rigorous discussion follows in Section 4. Finally, Section 5 concludes this paper.

## 2. Materials and Methods

The following section is divided into three subsections. Section 2.1 deals with Pepper’s hardware v1.8 (manufactured by Aldebaran Robotics, Paris, France, formerly SoftBank Robotics, Tokyo, Japan, now part of United Robotics, Bremmen, Germany), cameras (OV5640, OmniVision, CA, USA), and LIDAR sensors (Pepperl Fuchs, Mannheim, Germany). Section 2.2 deals with the OAs employed, such as PSO, the genetic algorithm, and GEO [18]. Section 2.3 introduces the iterative search strategy called the avoidance of local minima (ALM).

### 2.1. Pepper Robot and Cameras

The Pepper robot is a semi-humanoid mobile robot that is equipped with numerous built-in functions and operates on its own operating system (OS). NAOqi-OS is based on a Linux distribution and is installed on Pepper’s integrated computer (Intel ATOM, Santa Clara, CA, USA); however, users are restricted from installing third-party applications directly on the OS and must rely on Pepper’s proprietary Software Development Toolkit (SDK). Therefore, users can employ different tools to program and control the robot, e.g., GUI Choreographe, Python SDK, Linux SDK with a ROS environment, etc. Pepper comes with many integrated sensors, such as LIDARs, IRs (Pepperl Fuchs, Mannheim, Germany), sonars, RGB cameras, and a 3D camera (Asus, Taipei, Taiwan), which allow safe navigation and movement in a workspace. In this paper, the particular focus is on two different sensor families: the robot’s RGB cameras that provide color 3D images with depth information (RGB-D) and three separate LIDARs (front, left, and right). The visual data from the 3D camera can be used for feature extraction and mapping of the workspace—for instance, for detecting walls and corners—while the LIDARs can be used for autonomous and absolute localization of the robot within the workspace. The following subsection describes the cameras.

#### 2.1.1. Cameras

**Figure 1:**
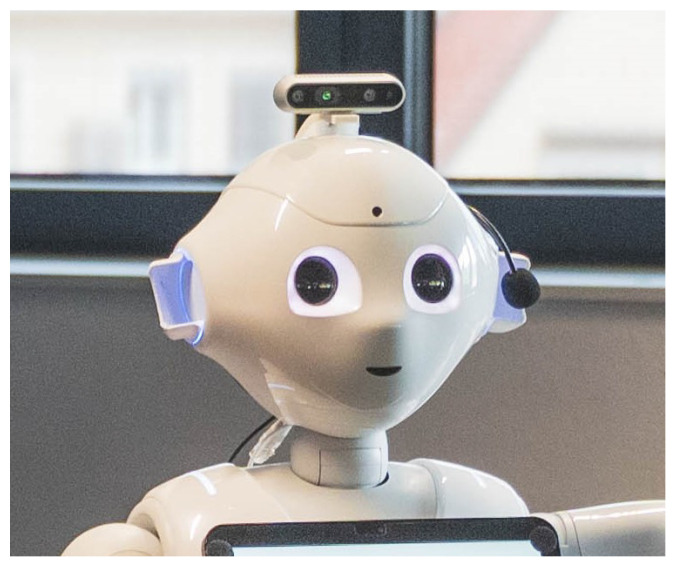
Pepper robot with a mounted RealSense D435 RGB-D camera on the top of the head. The video was streamed using Raspberry Pi 3.

Pepper’s cameras are preinstalled in the chin and in the forehead. They provide several adjustable resolutions for image processing tasks such as V-SLAM, although it was figured out that resolutions set too high become a problem due to the stalling WiFi connection. Experimentally, the image resolution at 320 × 240 pixels with 15–20 fps was realized as the maximum allowed, although the mapping results were very poor with higher signal-to-noise ratios. Selection of such a low resolution was recognized as insufficient; hence, we tested different external cameras to evaluate the usability, performance, and speed of camera image processing in ROS. A classic USB mono cam (Logitech HD C270 webcam, Lausanne, Switzerland) and RGB-D cameras (Kinect, RealSense D435, Cupertino, CA, USA and Luxonis DepthAI OAK-D Pro, Denver, CO, USA) were used in the visual SLAM tests while changing settings (resolution, fps, and Oriented FAST and Rotated BRIEF (ORB) features) and calibration parameters (camera distortion, low contrast, etc.). We were aiming for a high-accuracy point cloud for the later workspace map definition needed for localization based on known area dimensions (walls, corners, etc.). Due to the good SDK library support in ROS and Pepper, we decided on a RealSense D435 RGB-D camera that allowed us to operate at an enhanced rate of 640 × 480 pixels at 20 fps. As it was an external camera, we mounted it on the top of the robot’s head for improved vision, as seen in Figure 1. Again, the RGB-D video stream was streamed using the Raspberry Pi 3 node (Cambridge, UK).

#### 2.1.2. LIDARs

**Figure 2:**
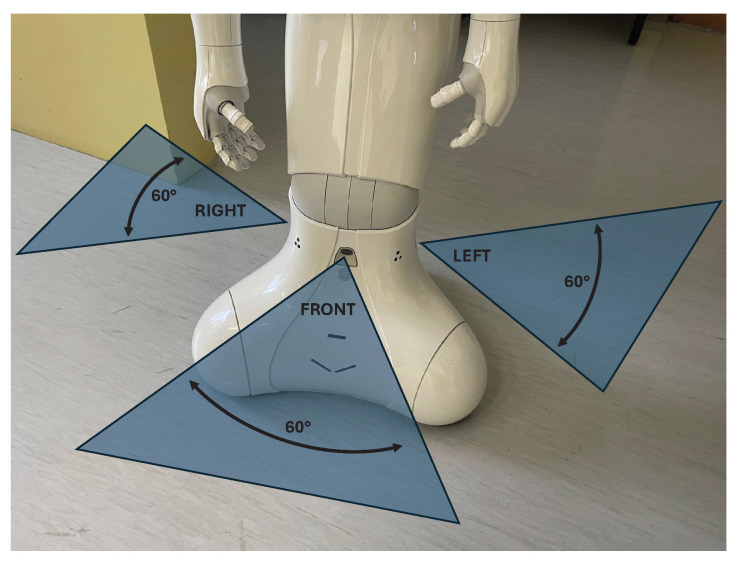
Locations and FOVs of the LIDARs on Pepper.

Three different LIDAR sensors are installed within Pepper. LIDAR sensors comprise a transmitting laser actuator and a separated receiving sensor. The sensors are offset by 90°, each with a horizontal field of view (HFOV, hereinafter field of view (FOV)) of 60°. To sense the whole workspace in 360°, Pepper needs either to be rotated or its LIDARs combined with other sensors. The layout of the LIDARs and their visible range is shown in Figure 2. The frame rate of each laser was 6.25 Hz, or 0.16 s. Each measured LIDAR section (front, left, and right) returns 15 segments divided into FOV = 4° with X and Y of the robot’s coordinate system. The accuracy of the measurements depends on the FOV angle, where segments more to the far left (segment 1) and far right (segment 15) are generally less accurate than the central segments. For example, central segment 8 was the most accurate. The built-in function returns the X and Y values of detected obstacles for each segment, which are then processed to calculate the angle and distance to the obstacle. Here, special cases exist regarding the uncertainty of the measurement accuracy, as the robot may return “Uncertain_Obstacle” or “Annoying_Reflection”. The analysis of these measurements showed no improvements in the measured distances but merely prolonged the data acquisition and processing times. A more detailed specification of the robot’s LIDARs can be found in Pepper’s documentation (http://doc.aldebaran.com/2-5/home_pepper.html, accessed on 12 June 2025).

The measurements were recorded with all three LIDARs of the robot. The workspace was realistic but artificially constructed with the help of wooden boards and the concrete walls of the laboratory. The workspace was measured precisely with a tape measure. The workspace had a starting point of the base coordinate system, upon which all other distances were derived to all the corners of the workspace in the picture. We placed the robot inside the defined workspace and performed real LIDAR measurements and compared them to the ground-truth measurements.

Additionally, we tested the accuracies of all three LIDARs by placing the robot in front of a flat wide wall, so that the center beam of the LIDAR (sensor segment 8, out of the total 15 segments) was perpendicular to the wall. We moved the robot away from the wall gradually and took measurements with the same LIDAR. The distance of the robot from the wall was additionally measured with a tape measure and a handheld laser meter. It was realized that the measurements deviated from the expected values. In addition, it was realized that the measurements were distorted in a curved way, even if measuring from a flat wall, and the distortion grew as the distance increased. The measurement of distances beyond 3.5 m was not useful practically.

#### 2.1.3. Robot’s Leg

**Figure 3:**
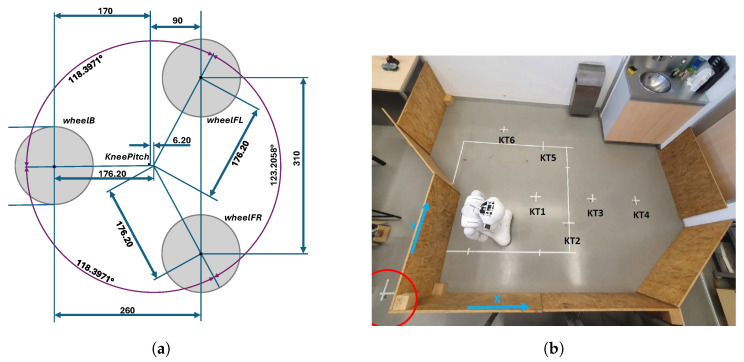
(**a**) Bottom side of the robot’s leg with three omnidirectional wheels. (**b**) Workspace built with OSB wooden boards.

The robot coordinate system has the X axis positive toward the robot’s front, the Y axis from right to left, and the Z axis vertical. Pepper is a semi-humanoid robot that moves using three sets of omnidirectional wheels (omni-wheels), i.e., a front left (FL), front right (FR), and back (B) wheel. These allow free movement in both X and Y directions as well as rotation around Z. The robot’s center of rotation is displaced by 6.2 mm from the KneePitch position, as seen from Figure 3 on the bottom view of the robot’s base.

#### 2.1.4. Test Workspace

Mobile robots move and navigate in a workspace according to the given map and a path plan. For evaluation of the proposed localization algorithm, a laboratory testing workspace with dimensions of 4 by 3.2 m was built with classic OSB wooden boards (the right plot of Figure 3). The surface of the workspace walls (boards) was selected as non-monothonic, so that the RGB-D camera was able to obtain proper results for the visual SLAM mapping and LIDAR measurements.

### 2.2. Algorithms and Methods Used

This section represents the PSO algorithm, the benchmarks, and the description of the implemented fitness function ff. Evolutionary and swarm intelligence OAs were selected instead of simple minimum gradient search algorithms due to future work considerations. Although gradient search would be sufficient for present tasks with lower dimensionalities (D < 4), we selected the more sophisticated OAs to ensure compatibility with anticipated future extensions, i.e., environments with larger dimensionalities (D ≥ 4), such as localization of airborne unmanned aerial vehicles.

#### 2.2.1. Particle Swarm Optimization

PSO is a population-based OA that was designed in 1995 by Kennedy and Eberhart [20]. PSO rests on the intelligent movement of a swarm that searches for prey by calculating consecutive velocity and position updates. It is an iterative optimization method, and its pseudocode is denoted in Algorithm 1. The canonical PSO was taken as a baseline, to which we added neighborhood best particle [21,22].
**Algorithm 1** Particle swarm optimization (PSO).  1: Initialize swarm with population size NP  2: Initialize positions $x_{i}$ and velocities $v_{i}$  3: Evaluate fitness function values $ff_{i}\;=\;fun\left(x_{i}\right)$  4: Set personal best $p_{i}\;=\;x_{i}$  5: Select global best $g\;=\;\mathrm{argmin}(f_{i})$  6: **while** termination condition not met **do**  7:    **for** each particle *i* in swarm NP **do**  8:      **for** each element *j* in dimension *D* **do**  9:         // Update velocity and position10:         $v_{i,t+1}^{\left(j\right)}\leftarrow c_{0}\cdot v_{i,t}^{\left(j\right)}\;+\;c_{1}\cdot r_{1}^{\left(j\right)}\cdot \left(p_{i,t}^{\left(j\right)}\;-\;x_{i,t}^{\left(j\right)}\right)\;+\;c_{2}\cdot r_{2}^{\left(j\right)}\cdot \left(g_{t}^{\left(j\right)}\;-\;x_{i,t}^{\left(j\right)}\right)\;+\;c_{3}\cdot r_{3}^{\left(j\right)}\left(l_{t}^{\left(j\right)}\;-\;x_{i,t}^{\left(j\right)}\right)$11:         $x_{i,t+1}^{\left(j\right)}\leftarrow x_{i,t}^{\left(j\right)}\;+\;v_{i,t}^{\left(j\right)}$12:      **end for**13:      Evaluate fitness $ff_{i}\;=\;ff\left(x_{i}\right)$14:      **if** $ff\left(p_{i}\right)\;<\;ff_{i}$ **then**15:         $p_{i}\leftarrow x_{i}$ Update personal best16:      **end if**17:      **if** $ff\left(g\right)\;<\;ff_{i}$ **then**18:         $g\leftarrow x_{i}$ Update global best19:      **end if**20:      **if** $ff\left(min\left(l_{i}^{*}\right)\right)\;<\;ff_{i}$ **then**21:         $l_{i}\leftarrow x_{i}${Update neighborhood best}22:      **end if**23:    **end for**24:    t←t + 125: **end while**26: **return** global best position g

The control parameters of PSO are inertia weight *w*, cognitive constant $c_{1}$, social constant $c_{2}$, and neighborhood constant $c_{3}$. The parameters $r_{1}$ and $r_{2}$ are random numbers drawn from the uniform distribution $r_{1},\;r_{2}\in \left[0,1\right]$. The personal best $p_{i}$ is updated when the particle itself obtains a better position. The global best g is updated when a particle, among all particles, obtains a better position. The $l_{i}^{*}$ represents the neighborhood. The neighborhood best $l_{i}$ is updated when a particle, among its neighborhood, obtains a better position. PSO was initialized with the parameter settings as outlined in Table 1 for both the simulation and laboratory experiments.

#### 2.2.2. Adaptive Elitist-Enhanced Genetic Algorithm (GA)

The adaptive elitist-enhanced GA was used as a benchmark. The genetic operators included roulette wheel selection, single-point crossover, bit-flip mutation, and elitism. Binary representation was implemented. An adaptive mutation factor $F_{m}$ was implemented to increase the mutation rate $p_{m}$ up to a certain value that equaled $p_{m}=0.05$ in the case of indicating no progress and reduce it down to a certain value that equaled $p_{m}=0.001$ in case of indicating progress, as follows in Equation (1). Here, $ff^{\left(avg\right)}$ represents the average value of the fitness function evaluations that were monitored for two different periods separately: First, for the last g−6 generations (*g* represents the current generation) to g−4 generations, i.e., $ff_{\left(g-6:g-4\right)}^{\left(avg\right)}$. Second, for the last g−3 to g−1 generations, i.e., $ff_{\left(g-3:g-1\right)}^{\left(avg\right)}$. Here, all the calculated fitness function evaluations were taken into account. The mutation rate $p_{m}$ was increased when the value $ff_{\left(g-3:g-1\right)}^{\left(avg\right)}$ did not exhibit any progress. When significant progress was indicated, the mutation rate was decreased. This mechanism decreased the probability of trapping into the local optima further. Algorithm 2 exhibits the GA pseudocode and Table 1 exhibits the GA setup.

(1) $$p_{m}=\left\{\begin{array}{cc} p_{m}\cdot F_{m} & ff_{\left(g-3:g-1\right)}^{\left(avg\right)}\in ff_{\left(g-6:g-4\right)}^{\left(avg\right)}\\ p_{m}/F_{m} & \mathrm{otherwise}. \end{array}\right.$$

**Algorithm 2** Adaptive elitist-enhanced genetic algorithm.  1: Initialize population NP with random solutions  2: Evaluate the fitness of each individual in NP  3: **while** stopping criterion not met **do**  4:    Convert population NP into binary form (genes)  5:    Select parents for reproduction  6:    Perform crossover and mutation to create offspring  7:    Convert binary genes of population NP back into decimal representation  8:    Evaluate the fitness of the offspring  9:    Apply elitism: select the best individuals for the next generation10:    Replace the worst individuals in the population with offspring11:    Apply probability of mutation $p_{m}$ adaptation12: **end while**

#### 2.2.3. Golden Eagle Optimization (GEO)

GEO is among the latest and most novel representatives of nature-inspired OAs. GEO resembles the spiral motion of golden eagles when cruising overhead the prey and, once finding the prey, attacking it. Cruise and attack effectively mirror the trade-off between the exploration and exploitation phases of swarm intelligence algorithms. The control parameters include the initial and final propensities to cruise and propensities to attack. The pseudocode is visualized in Algorithm 3, and the control parameter setup is shown in Table 1.
**Algorithm 3** Pseudocode of golden eagle optimizer (GEO).  1: Initialize the population of golden eagles  2: Evaluate fitness function  3: Initialize population memory  4: Initialize $p_{a}^{\left(GEO\right)}$ and $p_{c}^{\left(GEO\right)}$  5: **for** each iteration *t* **do**  6:    Update $p_{a}^{\left(GEO\right)}$ and $p_{c}^{\left(GEO\right)}$  7:    **for** each golden eagle *i* **do**  8:      Randomly select a prey from the population’s memory  9:      Calculate attack vector A10:      **if** length of attack vector A≠0 **then**11:         Calculate cruise vector C12:         Calculate step vector Δx13:         Update position: $x_{i}=x_{i}+\Delta x$14:         Evaluate fitness function for the new position15:         **if** fitness is better than the position in eagle *i*’s memory **then**16:           Replace the position in eagle *i*’s memory with the new position17:         **end if**18:      **end if**19:    **end for**20: **end for**21: Return the best solution

#### 2.2.4. Description of the Fitness Function

**Figure 4:**
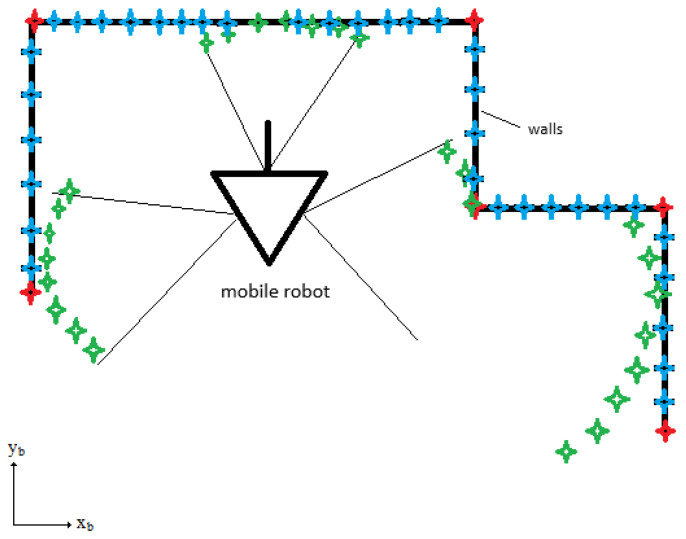
Architecture of the robot’s workspace. The black lines define walls of the workspace, red diamonds represent the ground truth corners, blue diamonds represent the equidistant positions on the walls and green diamonds represent the measured distances from the robot to the wall.

The fitness function value to evaluate the quality of each particle or individual during the execution of the OA is denoted as ff. Calculation of the fitness function consists of two steps. First, the fitness function $ff_{j}$ for each measured position *j* is calculated as the distance between $\left(x_{mj},\;y_{mj}\right)$ and the closest equidistant position on the wall $\left(x_{wi},\;y_{wi}\right)$, as described in Equation (2). Next, an average of these fitness functions is calculated as a final fitness function ff, as described in Equation (3)

(2) $$ff_{j}=\sqrt{{\left(x_{wi}-x_{mj}\right)}^{2}+{\left(y_{wi}-y_{mj}\right)}^{2}},$$

(3) $$ff=\frac{\sum_{j=1}^{m}ff_{j}}{m},$$

where i=1,…,n and j=1,…,m, *n* represents the number of equidistant positions on the walls between the corners of the mobile robot workspace in the base coordinate system, and *m* represents the number of positions measured by LIDAR in the base coordinate system. $x_{mj}$ and $y_{mj}$ represent positions measured by the LIDAR in the base coordinate system, as denoted in Figure 4. $x_{wi},\;y_{wi}$ represent equidistant positions on the walls between the corners of the mobile robot workspace in the base coordinate system, as denoted in Figure 4.

Figure 4 shows the organization of the workspace. The coordinate axes ($x_{b}$, $y_{b}$) define the base coordinate system. The red diamonds denote the corners of the workspace. The blue diamonds denote equidistant positions on the wall $x_{wi},\;y_{wi}$. The green diamonds denote measured distances between the mobile robot and the walls by the LIDAR $x_{mj},\;y_{mj}$. A triangle and a forward path line define the location and orientation of the mobile robot. The black lines define the walls of the workspace.

### 2.3. Algorithm for Avoiding the Local Minima (ALM)

The scientific literature has often criticized PSO for not having any global optima avoidance mechanism. Especially if relying on the gbest action that may cause premature convergence and not having proper momentum settings, PSO can be trapped in the local minima easily [23]. This problem is usually solved with repeated dispersion of particles from the randomly chosen initial positions, even several times if needed. Knowing the global minimum helps significantly in achieving it, although, in the majority of the cases, the global optimum is not known in practice. In our case, the fitness function value of the global minimum was not known directly. Furthermore, due to the noise of the LIDAR used, a solution in the “spurious” local minima may have the same or, in rare cases, even better fitness function value than in the “truly” global minimum. This means that we cannot estimate directly if the OA, in general, is trapped or not. However, the integrity of the solution can be checked indirectly by moving the robot for a known distance and rotation (the absolute value of the difference betweem the reference position and reference rotation value and the previous, already calculated, actual position and rotation) and making another measurement, hence comparing two consecutive measurements effectively. If the integrity check returns nonsense with regard to the Markov chain, this is then a reliable indicator that the OA is trapped in the local minima.

To cope with this, the classical OAs were tailored to address the challenge of local optima effectively. The idea of avoiding the local minima in the PSO, GA, and GEO algorithms is not a general solution, but rather an application-oriented solution. It is valid only for the localization of the mobile robot application or very similar applications with low signal-to-noise ratios. The odometrical method measures the distance traveled and orientation shifted between two relatively close positions of the mobile robot accurately. For longer distances traveled, the odometrical method is not a reliable indicator due to the omni-wheel slip and constantly accumulating error (in particular the rotation in the Z axis causes odometrical challenges). However, for shorter distances of up to two meters of linear travel and a rotation of less than 90°, the odometrical method works just fine, imposing just a few centimeters of error between two consecutive movements.

Practically, it was realized that the distance error margin of Δp<15 cm and the rotation error margin of Δω<15°, due to omni-wheel slip and accumulating error, resemble the sufficient error margins based on practical observations.

Meeting these two error margins simultaneously means passing the integrity check, meaning that the global minimum was found. When the OA does not find the global optimum within a given maximum number of iterations or generations, the algorithm is reset and run again. The so-called avoiding local minima search strategy (ALM) pseudocode is exhibited in Algorithm 4. If both the error margins are met, the integrity check is passed and the global best solution is returned. When the error margins are not met, the OA is repeated by dispersing the particles or individuals again in a while loop. The process is repeated until the testing solution meets the imposed error margins, which, unfortunately, imposes potential time delays.

The ALM search strategy commences by initializing the particles or individuals randomly. The variable repeated monitors the number of OA resets after not finding the global optima. It is set to 0. The variables $p_{r1}$ and $\omega_{r1}$ measure the unknown initial reference position and orientation. The variables $p_{r2}$ and $\omega_{r2}$ measure the unknown ending reference position and orientation. The differences $\Delta p_{r}$ and $\Delta \omega_{r}$ measure the reference move.

**Figure 5:**
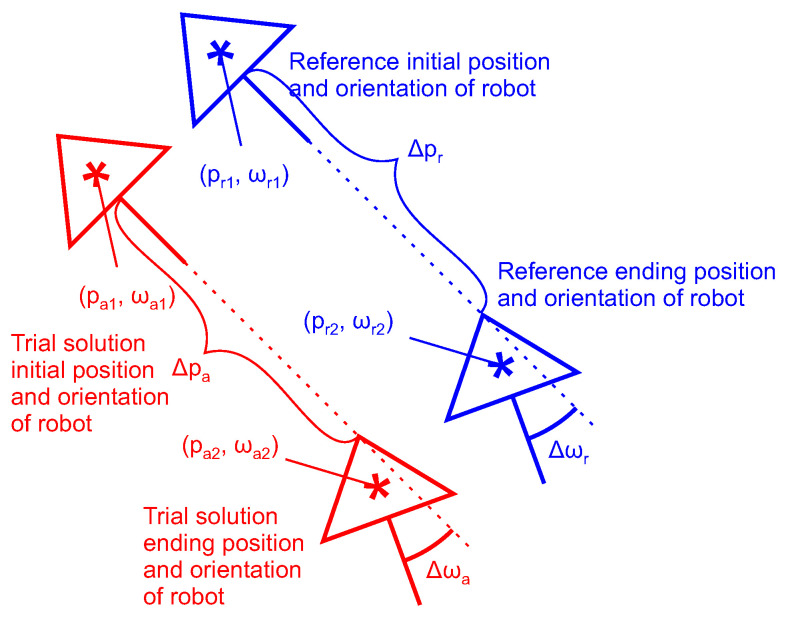
Notation for robot’s poses. The blue symbols and lines represent the reference positions and orientations. The red symbols and lines represent the trial solution positions and orientations by the OA.

The variables $p_{a1}$ and $\omega_{a1}$ indicate an OA trial solution for initial position and orientation, where the trial solution means each particle or individual in every epoch or generation. The variables $p_{a2}$ and $\omega_{a2}$ indicate an OA trial solution for the ending position and orientation. The differences $\Delta p_{r}$ and $\Delta \omega_{r}$ measure the reference move. The differences $\Delta p_{a}$ and $\Delta \omega_{a}$ indicate a move performed for the given trial solution (see Figure 5).

The procedure for the ALM search strategy is as follows: First, the robot is placed at an unknown position $p_{r1}$ within the predetermined search space. Here, the LIDAR measurements are performed. Next, the robot is moved to position $p_{r2}$. Again, the LIDAR measurements are performed and recorded. The reference distance and rotation change are calculated as follows: $\Delta p_{r}=\left|p_{r1}-p_{r2}\right|$ and $\Delta \omega_{r}=\omega_{r1}-\omega_{r2}$.

Then, the OA is executed to calculate and evaluate the trial solutions. The best solutions $p_{a1}$ and $\omega_{a1}$ are output for the initial position and $p_{a2}$ and $\omega_{a2}$ for the ending position. These are then compared to calculate the distance and orientation errors Δp and Δω. As long as these two fall outside the allowed error margins, the OA is rerun randomly again. The variable repeated is incremented with each rerun. Once the errors meet the error margins, the global best solutions $\left(p_{gbest_{1}},\omega_{gbest_{1}}\right)$ and $\left(p_{gbest_{2}},\omega_{gbest_{2}}\right)$ are output.

The ALM search strategy pseudocode is exhibited in Algorithm 4.
**Algorithm 4** The ALM initial search strategy pseudocode.  1: Move the robot to $\left(p_{r1},\omega_{r1}\right)$  2: Measure the LIDAR data  3: Move the robot to $\left(p_{r2},\omega_{r2}\right)$  4: Measure the LIDAR data  5: Input: reference distance and rotation $\left(\Delta p_{r},\Delta \omega_{r}\right)$  6: repeated←0  7: Calculate: optimization algorithm result $\left(p_{a1},\omega_{a1}\right)$  8: Calculate: optimization algorithm result $\left(p_{a2},\omega_{a2}\right)$  9: $\Delta p_{a}\leftarrow \left|p_{a1}-p_{a2}\right|$10: $\Delta \omega_{a}\leftarrow \left|\omega_{a1}-\omega_{a2}\right|$11: $\Delta p\leftarrow \left|\Delta p_{a}-\Delta p_{r}\right|$12: $\Delta \omega \leftarrow \left|\Delta \omega_{a}-\Delta \omega_{r}\right|$13: **while** Δp>15 cm and Δω>15° **do**14:    Calculate: optimization algorithm result $\left(p_{a1},\omega_{a1}\right)$15:    Calculate: optimization algorithm result $\left(p_{a2},\omega_{a2}\right)$16:    $\Delta p_{a}\leftarrow \left|p_{a1}-p_{a2}\right|$17:    $\Delta \omega_{a}\leftarrow \left|\omega_{a1}-\omega_{a2}\right|$18:    $\Delta p\leftarrow \left|\Delta p_{a}-\Delta p_{r}\right|$19:    $\Delta \omega \leftarrow \left|\Delta \omega_{a}-\Delta \omega_{r}\right|$20:    repeated←repeated+121: **end while**22: $p_{gbest1},\omega_{gbest1},p_{gbest2},\omega_{gbest2}\leftarrow$ global best solutions for both poses23: **return** $p_{gbest1},\omega_{gbest1},p_{gbest2},\omega_{gbest2}$

If both the error margins are met, the integrity check is passed. Then, the two poses of $\left(p_{gbest1},\omega_{gbest1}\right),\left(p_{gbest2},\omega_{gbest2}\right)$ are output from the ALM as a global minimum solution. If the error margins are not met, the search is repeated by dispersing the particles or individuals again until the testing solution meets the imposed error margins. In addition, the OA needs to be run twice for both unknown first $\left(p_{a1},\omega_{a1}\right)$ and second robot positions $\left(p_{a2},\omega_{a2}\right)$. However, for each subsequent position, e.g., a third position or $\left(p_{a,i+1},\omega_{a,i+1}\right)$, in general, just a single OA run is necessary, as the *i*-th position is known. Therefore, Algorithm 4 was modified slightly, as follows in Algorithm 5. Accordingly, the average overall elapsed time $\bar{t_{o}}$ has been reduced by more than twice.
**Algorithm 5** The ALM search strategy pseudocode for each subsequent robot position.  1: Move the robot to $\left(p_{r,i+1},\omega_{r,i+1}\right)$  2: Measure the LIDAR data  3: Input: reference distance and rotation $\left(\Delta p_{r}^{\left(i+1\right)},\Delta \omega_{r}^{\left(i+1\right)}\right)$  4: repeated←0  5: Calculate: optimization algorithm result $\left(p_{a,i+1},\omega_{a,i+1}\right)$  6: $\Delta p_{a}\leftarrow \left|p_{a,i}-p_{a,i+1}\right|$  7: $\Delta \omega_{a}\leftarrow \left|\omega_{a,i}-\omega_{a,i+1}\right|$  8: $\Delta p\leftarrow \left|\Delta p_{a}-\Delta p_{r}\right|$  9: $\Delta \omega \leftarrow \left|\Delta \omega_{a}-\Delta \omega_{r}\right|$10: **while** Δp>15 cm and Δω>15° **do**11:    Calculate: optimization algorithm result $\left(p_{a,i+1},\omega_{a,i+1}\right)$12:    $\Delta p_{a}\leftarrow \left|p_{a,i}-p_{a,i+1}\right|$13:    $\Delta \omega_{a}\leftarrow \left|\omega_{a,i}-\omega_{a,i+1}\right|$14:    $\Delta p\leftarrow \left|\Delta p_{a}-\Delta p_{r}\right|$15:    $\Delta \omega \leftarrow \left|\Delta \omega_{a}-\Delta \omega_{r}\right|$16:    repeated←repeated+117: **end while**18: $p_{gbest,i+1},\omega_{gbest,i+1}\leftarrow$ global best solution for the i+1 pose19: **return** $p_{gbest,i+1},\omega_{gbest,i+1}$

### 2.4. Mapping of the Workspace by the ORB-SLAM2 Method and RANSAC Algorithm

The Pepper robot has 2D and 3D cameras, which means that applying V-SLAM for initial mapping of the workspace was natural. Although robot LIDARs are adequate for mapping surfaces near the ground, Pepper must also be careful of higher obstacles while navigating to prevent falling. Pepper’s native 3D camera assures the security perimeter, so the robot stops before hitting the obstacle, but for SLAM tasks the results can be poor. Therefore a RealSense RGB-D camera was used with ORB-SLAM2, mounted on Pepper’s head, for enhanced point cloud retrieval and a more detailed workspace map.

ORB-SLAM2 [24] rests on detecting distinct features that are input into the mapping algorithm. It is designed for real-time applications in robotics and computer vision. Developed as an extension of ORB-SLAM, it offers improvements in robustness, efficiency, and adaptability across monocular, stereo, and RGB-D camera inputs. The algorithm utilizes ORB features for keypoint detection and descriptor matching. ORB features are computationally efficient and invariant to rotation and scale, making them well-suited for real-time SLAM applications. The algorithms can be used with monocular cameras, which require a motion-based initialization, while stereo and RGB-D cameras can compute a scaled map directly from depth information. The algorithm estimates the camera pose continuously by matching the extracted ORB features with keypoints in previous frames, and it employs a motion model to predict the next pose and refines estimates using bundle adjustment. It maintains a local map of the workspace by adding new keyframes and refining 3D poses using local bundle adjustment. This process ensures accurate tracking and prevents drift in the estimated trajectory. Finally, to prevent long-term drift, ORB-SLAM2 detects loop closures by matching features from the current frame with previously visited locations. Once a loop closure is detected, the system performs pose graph optimization to correct any accumulated errors.

The ORB-SLAM2 algorithm was not performed on the robot due to its CPU overload in the case of such high processing demand. Rather, the algorithm was executed on an Intel i7 laptop using 16 GB of RAM, running Ubuntu 16.04 LTS with ROS Kinetic. The ORB-SLAM2 used a RealSense D435 camera in RGB-D mode with a properly set up configuration to obtain optimal features for mapping. A RANSAC algorithm was used to be able to recognize and model a workspace with walls and corners from a previous gathered map (point cloud and features).

RANSAC is the short name for the Random Sample Consensus Algorithm. It is used for finding the best fit function for an existing dataset with outliers. Using RANSAC, datasets could be fitted to a single variable, as well as multivariable functions. In comparison to the least squares method (LSM) for finding the best fit function, it is more robust to outliers. RANSAC removes outliers from datasets iteratively before fitting the function to the data by the LSM or some other M-estimator algorithm. To remove outliers, RANSAC samples a part of the points from the existing dataset randomly and fits the function to this sampled dataset. Next, the absolute error between the function and randomly sampled points is calculated, and the points with the greatest error are considered as outliers and are removed from the dataset. Random sampling, error calculation, and elimination of outliers are iterated until random sampling of points from the dataset does not produce any change in the parameters of the function fitted to the dataset. Many improvements of the RANSAC algorithms have been proposed, and among them is MLESAC [25].

## 3. Results

The experiments were divided into two large segments: simulation experiments and real-world laboratory (lab) experiments. The simulation experiments were performed first and involved proving the concept. Once the concept was proved, the real lab experiments were performed.

### 3.1. Conduct of Experiments and Experimental Setup

The conduct of the simulation experiments was as follows. First, the MATLAB/Simulation environment was used to design the exact workspace as in a real lab experiment. Then, the model of the LIDAR measurements was input. Different qualities of the simulated LIDAR measurements and workspace corners were imposed, ranging from the most accurate to least accurate by employing uniform random perturbations. Perturbation of the LIDAR measurements represents the errors imposed by LIDAR. Perturbation of the workspace corners represents the errors imposed by the mapping algorithm (ORB-SLAM2 and RANSAC), since the latter also imposes deviations from the ground truth. The OAs were then executed, and their performance was monitored closely. The OAs’ control parameters were set as outlined in Table 1.

The conduct of the real lab experiments was as follows. A laboratory workspace was built with Pepper put into it. The first set of the LIDAR measurements were taken. Then, Pepper was moved for a certain distance and rotation. Next, a second set of LIDAR measurements were taken. Then, the OA was executed on the remote computer to calculate Pepper’s position.

### 3.2. Simulation Experiments

The simulation experiments were run completely in the MATLAB/Simulink environment with the objective of (1) modeling the LIDAR errors and modeling the RANSAC workspace corners’ definition errors and (2) realizing the underlying OA performance. Such simulation experiments were needed to estimate the influence of both qualities of the real mobile robot LIDAR measurements and the workspace corner measurements, helping improve the quality of OA performance in calculating position and orientation.

**Figure 6:**
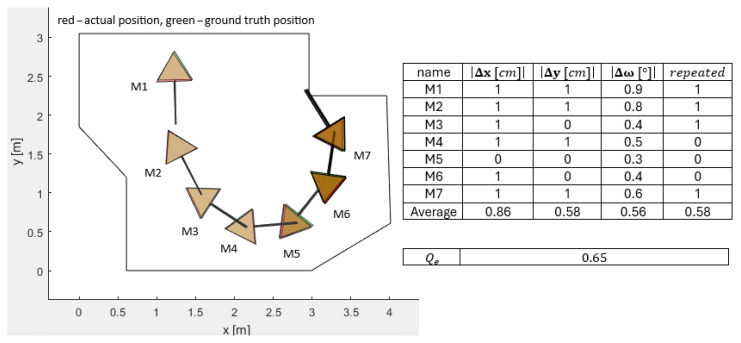
Simulated differences of the actual and ground-truth positions and the orientations for the LIDAR accuracy measurements $pc_{L}=\pm 1\%$ and the accuracy measurements of the workspace corners $pc_{c}=\pm 1\%$.

**Figure 7:**
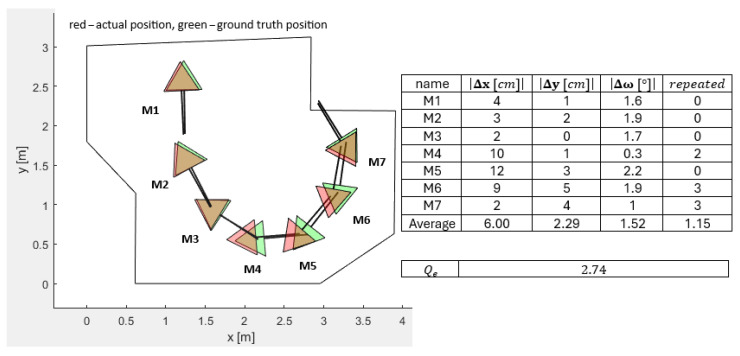
Simulated differences of the actual and ground-truth positions and orientations for the LIDAR accuracy measurements $pc_{L}=\pm 1\%$ and the accuracy measurements of the workspace corners $pc_{c}=\pm 5\%$.

**Figure 8:**
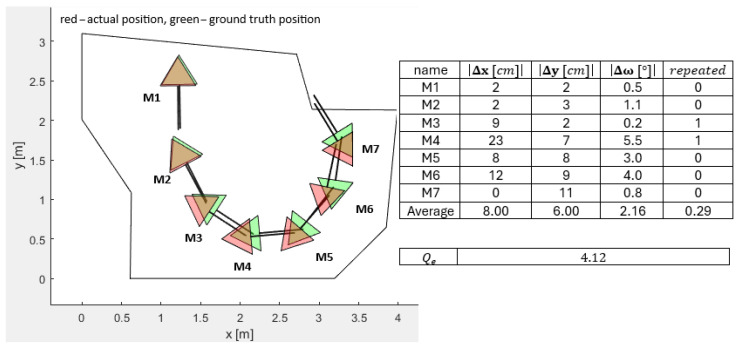
Simulated differences of actual and ground-truth positions and orientations for LIDAR accuracy measurements $pc_{L}=\pm 1\%$ and accuracy measurements of workspace corners $pc_{c}=\pm 10\%$.

**Figure 9:**
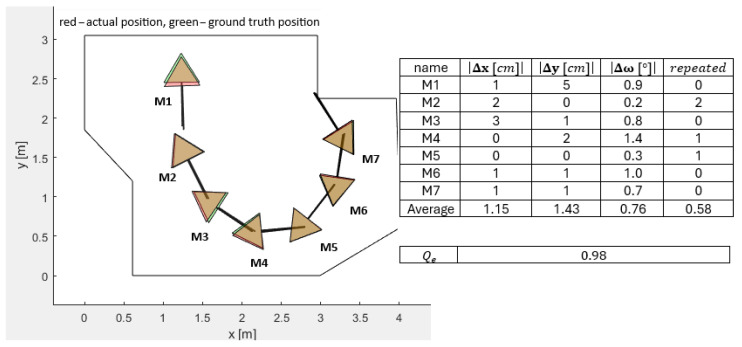
Simulated differences of the actual and ground truth positions and orientations for the LIDAR accuracy measurements $pc_{L}=\pm 5\%$ and the accuracy measurements of the workspace corners $pc_{c}=\pm 1\%$.

**Figure 10:**
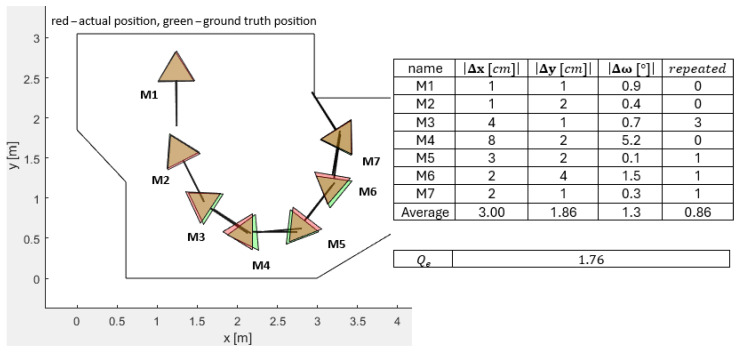
Simulated differences of the actual and ground-truth positions and orientations for the LIDAR accuracy measurements $pc_{L}=\pm 10\%$ and the accuracy measurements of the workspace corners $pc_{c}=\pm 1\%$.

Figure 6, Figure 7, Figure 8, Figure 9 and Figure 10 represent the simulation experiments of different magnitudes of error perturbation. Representation of the the robot’s position and orientation is consistent throughout this paper. The red and green triangles present the two-dimensional footprint of Pepper. The straight black lines present the orientation of the mobile robot. The green triangles represent the simulated ground-truth position and orientation. The red triangles represent the simulated position and orientation executed by the OA. Seven different mobile robot positions and orientations, i.e., M-poses, e.g., M1 to M7, are drawn in each Figure 6, Figure 7, Figure 8, Figure 9 and Figure 10. These M-poses represent the simulated path that the Pepper model underwent. For each M-pose, the following deviations can be calculated: (1) the absolute difference between the actual simulated positions and orientations and ground truth (|Δx|, |Δy|, |Δω|), (2) an indicator of the OA repetitions (random dispersions, i.e., an indicator of OA trapping), and (3) the value of the quality function of the solution $Q_{e}$, where lower values of $Q_{e}$ represent a statistically better quality of the results. The LIDAR errors were modeled by the random perturbation parameter, where different magnitudes of the perturbations were implemented. Two types of uniform random perturbations were modeled, i.e., the different qualities of the LIDAR measurements $pc_{L}=\left\{\pm 1\%,\pm 5\%,\pm 10\%\right\}$, as well as different measurements of the test robot workspace corners $pc_{c}=\left\{\pm 1\%,\pm 5\%,\pm 10\%\right\}$. Here, $v_{i}^{\left(c\right)}={\left[v_{i,x}^{\left(c\right)},v_{i,y}^{\left(c\right)}\right]}^{T}$ represents the set of workspace corners in *x* and *y* axes. Additionally, $v_{i}^{\left(L\right)}={\left[v_{i,x}^{\left(L\right)},v_{i,y}^{\left(L\right)}\right]}^{T}$ represents the simulated LIDAR measurements. These undergo the random perturbations as denoted in Equation (4)

(4) $$v_{i}^{\left(pc\right)}=\begin{array}{c} v_{i}^{\left(c\right)}-v_{i}^{\left(c\right)}\cdot pc_{c}\cdot \frac{\mathrm{rand}(2,1)}{100}, \end{array}$$

if simulating LIDAR errors, or alternatively in Equation (5) if simulating the LIDAR measurements

(5) $$v_{i}^{\left(pL\right)}=\begin{array}{c} v_{i}^{\left(L\right)}-v_{i}^{\left(L\right)}\cdot pc_{L}\cdot \frac{\mathrm{rand}(2,1)}{100}, \end{array}$$

where $v_{i}^{\left(pc\right)}$ represents the perturbed corners and $v_{i}^{\left(c\right)}$ stands for unperturbed measurements (ground truth) of the robot’s workspace, given in a Cartesian coordinate system (c.s.) in polar form (r,θ) to be transformed into Cartesian form. Additionally, $v_{i}^{\left(pL\right)}$ represents the perturbed LIDAR measurements. The $pc_{L},pc_{c}=\left\{\pm 1\%,\pm 5\%,\pm 10\%\right\}$ represent percentage values of the LIDAR measurement perturbation or the percentage value of the perturbations of the definition of the corners of the workspace. rand(2, 1)∈[−1, 1] represents the two-dimensional ${\left(x,y\right)}^{T}$ random value drawn from the uniform distribution. The process of perturbing the LIDAR measurements and workspace corners is as follows.

First the workspace corners are perturbed, given the ground truth $v_{i}^{\left(c\right)}$. Here, each ${\left(x,y\right)}^{T}$ component of each workspace corner is perturbed randomly for an amount equal to $pc_{c}\cdot rand$. This creates perturbed workspace corners $v_{i}^{\left(pc\right)}$. Next, based on the positions of perturbed corners, line segments from one corner to another are drawn. Based on these, LIDAR apparent ground-truth measurements are derived $v_{i}^{\left(L\right)}$ mathematically. Finally, the apparent ground-truth measurements $v_{i}^{\left(pL\right)}$ are perturbed for an amount equal to $pc_{L}\cdot rand$.

Furthermore, the quality of OA solutions is calculated using the quality function $Q_{e}$, which is calculated as denoted in Equation (6).

(6) $$Q_{e}=\left(\frac{\sum_{i=1}^{7}\left|\Delta x\right|}{7}+\frac{\sum_{i=1}^{7}\left|\Delta y\right|}{7}+\frac{\sum_{i=1}^{7}\left|\Delta \omega \right|}{7}+\frac{\sum_{i=1}^{7}repeated}{7}\right)/4,$$

where $\left|\Delta x\right|$ presents the absolute error between the actual position and the ground-truth position in the *x* axis of the mobile robot, $\left|\Delta y\right|$ presents the absolute error between the actual position and the ground-truth position in the *y* direction of the mobile robot, $\left|\Delta \omega \right|$ presents the absolute error between the actual orientation and ground-truth orientation of the mobile robot, and repeated represents the number of OA dispersions when finding the global minimum. For a sanity check, two evaluations of the quality function value $Q_{e}$ were performed as follows:

- LIDAR measurements perturbed $pc_{L}=\pm 5\%$ and the corners perturbed $pc_{c}=\pm 5\%$: $Q_{e}=2.25$;
- LIDAR measurements perturbed $pc_{L}=\pm 10\%$ and the corners perturbed $pc_{c}=\pm 10\%$: $Q_{e}=4.26$.

### 3.3. Practical Laboratory Experiments

**Figure 11:**
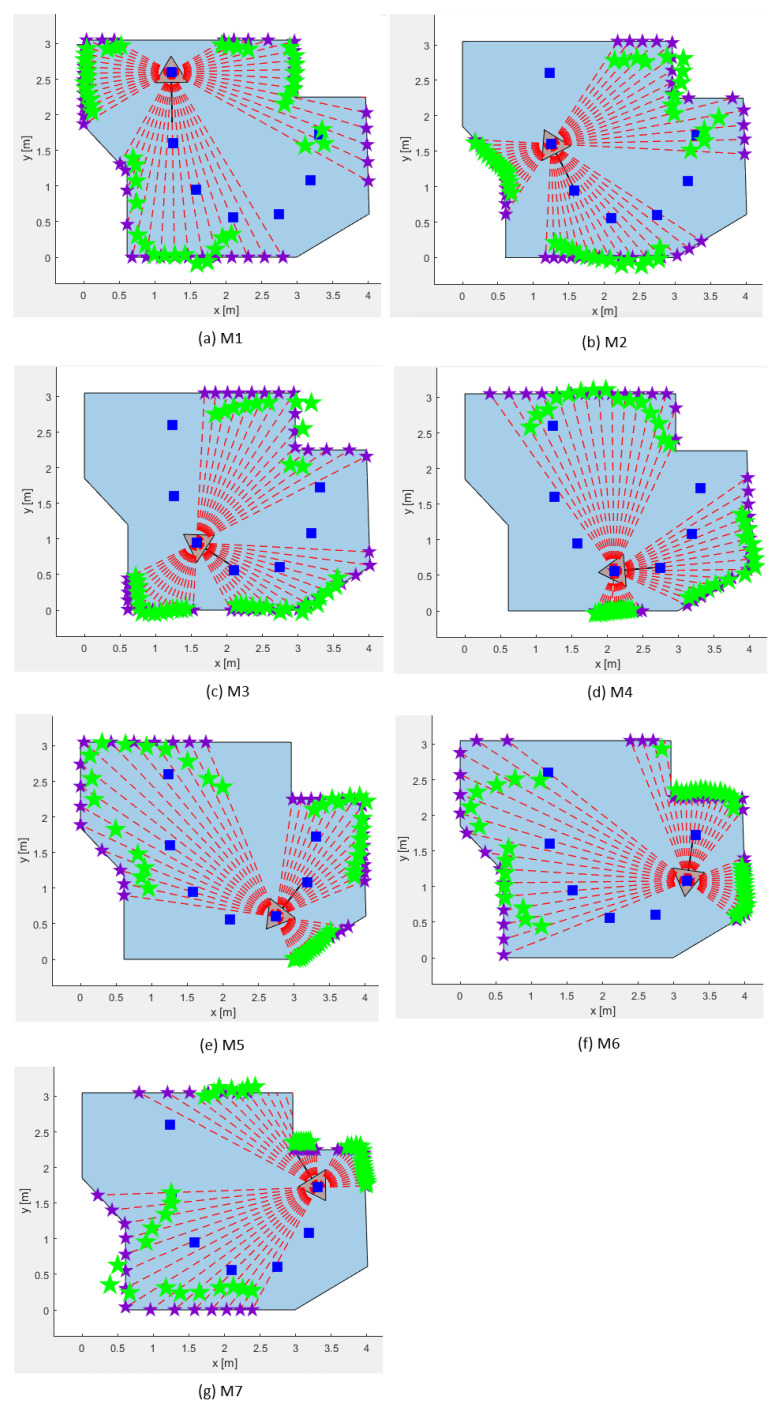
LIDAR measurements in the real lab experiment: green stars—real measurements; purple stars—prediction of exact measurements.

The real laboratory LIDAR measurements on the mobile robot Pepper exposed a significant drawback; i.e., the LIDAR measurements were of severely low quality. The low-quality LIDAR measurements for all M-poses can be observed in Figure 11. The purple stars mark the ideal distance measured from the robot to the edges of the workspace. The green stars mark the actual distance measured by the LIDAR. The deviation between the two represents the error. Larger errors were realized (1) for the outer edges of the LIDAR and (2) when increasing the distance to the wall. Hence, the laser beam scanning width (FOV) was limited to 44° and the maximum measured distance was limited below 3.5 m.

#### 3.3.1. The Plain Vanilla PSO Benchmark and Representation of the Local Optima Trapping

**Figure 12:**
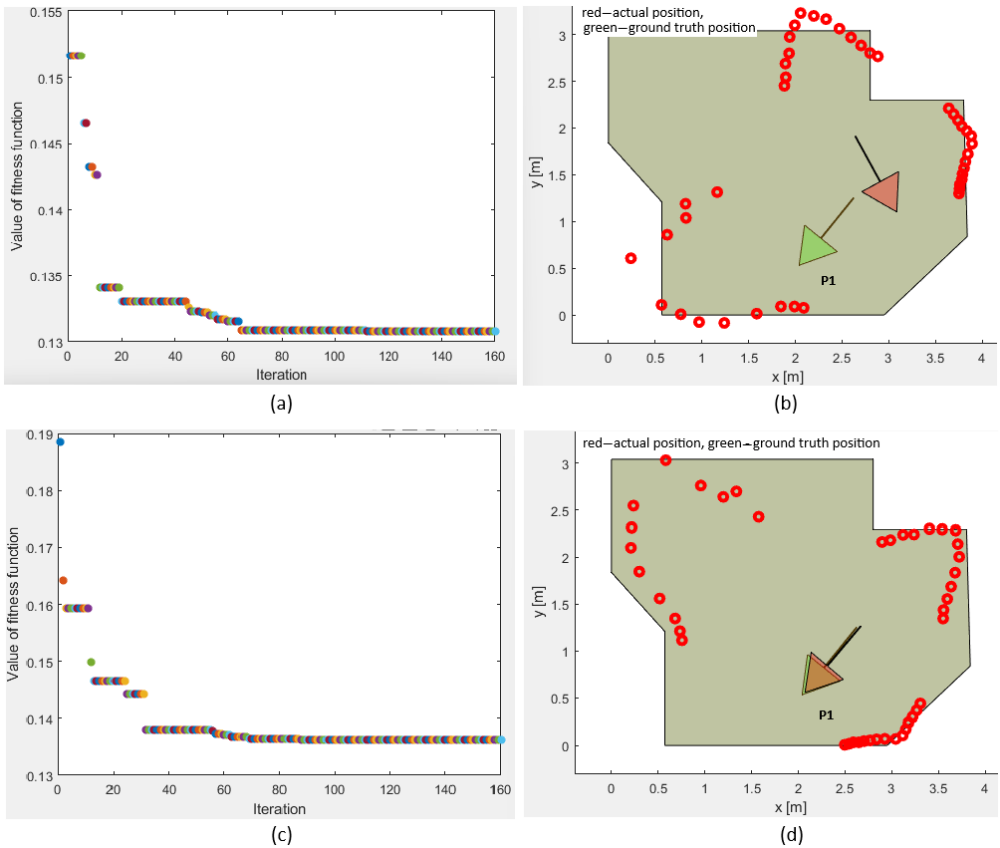
Calculating the position and orientation with the plain vanilla PSO. Subfigure (**a**) represents converging of the fitness function value for a misidentifies robot’s location; (**b**) an example of misidentified robot’s location, i.e., ground truth position in green, calculated position in red; (**c**) converging of the fitness function value for a well identified robot’s location; (**d**) an example of well identified robot’s location, i.e., ground truth position in green, calculated position in red, thus red covering the green triangle.

Within the workspace there are several different locations that undergo similar solutions. The low accuracies of the LIDAR could favor several possible locations (local optima) rather than the ground-truth location (global optimum), thereby imposing multiple local optima. Such a location is the pose P1, denoted in Figure 12. The pose P1 was used in a plain vanilla PSO experiment. The objective of this experiment was to examine whether the plain vanilla PSO could trap into the local optima. The experiment was carried out by executing PSO just once, not including the implemented local optima avoidance mechanism. The whole procedure was run multiple times to realize potential local optima. Figure 12 represents the two possible results that PSO statistically favored. In the upper subplot, the mobile robot was trapped into the local optimum, causing significant deviation of the measured location from the ground truth. The lower subplot includes the identical set of LIDAR measurements. PSO was run again from the beginning. Now, the global optimum was found, thus decreasing the deviation from the ground truth significantly. Further analysis exhibited that the local optima scored a lower fitness function value ff than the global optimum, contributing to the complexity of the challenge and expressing the necessity for the local optima avoidance mechanism.

#### 3.3.2. Quality Function Value Convergence for Pose P1 $Q_{e-P1}$ with Respect to the Population Size NP and Number of Iterations $N_{i}$

**Figure 13:**
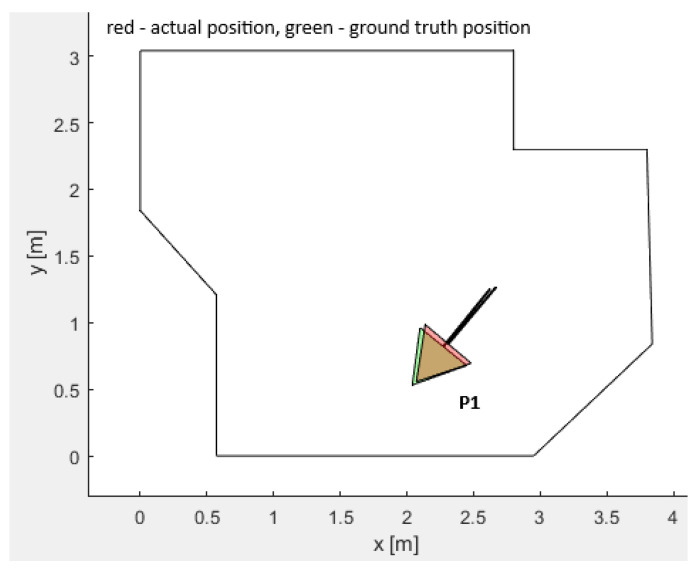
The P1 localization for which the influence of the number of iterations $N_{i}$ and population size NP on the enhanced quality function value was calculated.

The number of iterations $N_{i}$ and the population size NP in the PSO algorithm with the algorithm to avoid local minima had an important influence on the elapsed time of the execution of the PSO algorithm and the quality of the calculated position and orientation of the mobile robot. The influence of $N_{i}$ and NP on the quality of the PSO was measured again at position P1 (Figure 13). The quality function value $Q_{e}$ introduced was tailored to a single pose, as denoted in Equation (7).

(7) $$Q_{e-P1}=\frac{\left|\Delta x\right|+\left|\Delta y\right|+\left|\Delta \omega \right|+\bar{repeated}}{4}$$

Table 2 shows the results of varying the number of iterations $N_{i}$ and the number of particles NP, while simultaneously monitoring the quality of the calculated positions and orientations at P1 and value $Q_{e-P1}$.

Further discussion of the convergence results is given in the Section 4.

#### 3.3.3. PSO Localization with ±1% Accurate Measured Corners of the Workspace (Handheld Method)

**Figure 14:**
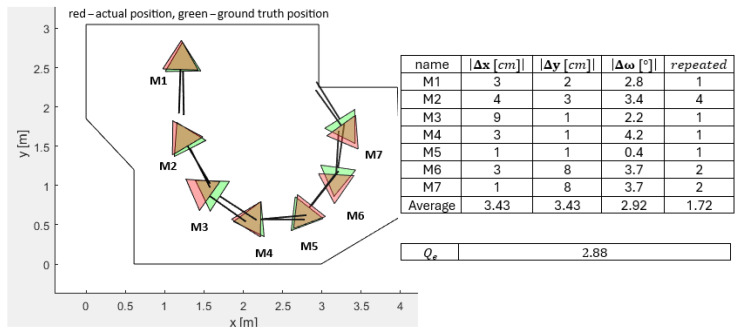
Differences of the real actual and ground-truth positions and orientations for the real LIDAR measurements and the estimated accuracy measurements of workspace corners +/−1%.

The first lab experiment with the PSO with the local optima avoidance mechanism was to prove its functioning. The experiment consisted of measuring the corners of the workspace, manually with a high degree of accuracy, giving the obtained map to the PSO and calculating the absolute positions of Pepper within this workspace. The real ground-truth positions (green) and orientations and the real actual positions (red) and orientations executed by the described PSO algorithm for all M-poses are presented in Figure 14. The real LIDAR measurements, as presented in Figure 11, were used for calculation of the positions and orientations of the mobile robot with the PSO algorithm. The manually measured corners of the workspace were estimated to be within ±1% accuracy.

An experiment of scanning with different FOV angles and monitoring quality function values and time elapsed simultaneously was performed in order to realize optimal LIDAR’s FOV scanning angle. Table 3 represents the results of different FOV angles. The average elapsed time of a single measurement and a PSO execution is denoted as $\bar{t_{e}}$. The average overall time of a single measurement and multiple PSO executions (no. of $\bar{repeated}$ times) in the case of trapping is denoted as $\bar{t_{o}}$. Equation (8) represents the formula for the average elapsed time and average overall time, as follows.

(8) $$\bar{t_{o}}=\bar{t_{e}}\cdot \bar{repeated}+\bar{t_{e}}$$

In general, the higher the FOV, the longer the average elapsed time of scanning $\bar{t_{e}}$. The higher the FOV, the longer the total overall time $\bar{t_{o}}$ on average. For the practical experiments, the FOV angle was limited to FOV = 44° as a compromise between the average quality function values $Q_{e}$ and time complexity $\bar{t_{o}}$.

#### 3.3.4. PSO Localization with a Corner Accuracy of 15% of the Workspace (ORB-SLAM2 and RANSAC Algorithm Method)

**Figure 15:**
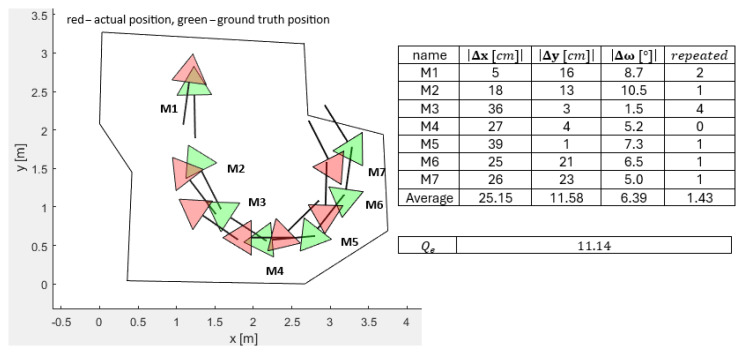
Differences of the real actual and ground-truth positions and orientations for the real LIDAR measurements and the workspace corners calculated by ORB-SLAM2 and RANSAC algorithms.

The next lab experiment consisted of the automated workspace corner measurements using the ORB-SLAM2 mapping methods and a RANSAC algorithm for corner extraction using the camera. The ground truth measured from the handheld method is not supplied here. The accuracy of the RANSAC algorithm workspace corner definitions was estimated as 15%. The diminished accuracy reproduced the increased random perturbation $pc_{c}$ parameter used in the simulations, which, naturally, complicated the challenge of absolute localization within the workspace. Figure 15 represents the typical workspace corner identification and related errors. The quality function value increased from $Q_{e}=2.88$ for the handheld method to $Q_{e}=11.14$ for the RANSAC, thus weakening the localization skills significantly.

#### 3.3.5. Influence of Obstacles in the Mobile Robot Workspace

**Figure 16:**
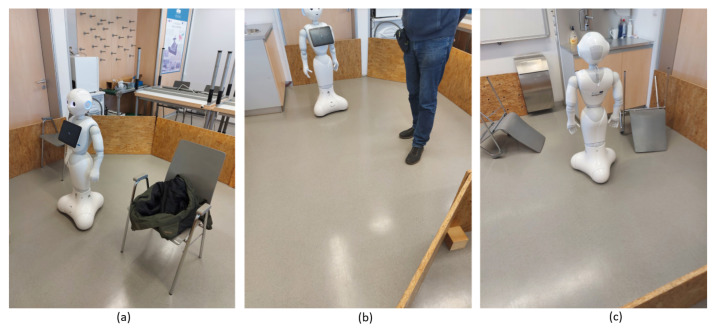
(**a**) Minor (small) obstacles, (**b**) medium obstacles, and (**c**) major (large, huge) obstacles.

Calculations of the positions and orientations of the mobile robot with obstacles inside of the robot’s workspace were performed by the PSO-ALM for three types of obstacles: minor obstacles (legs of the chairs), medium obstacles (human feet) and major obstacles (overturned chair) (see Figure 16).

The minor obstacles did not cause additional position and orientation errors for the PSO-ALM. This was due to the fact that the number of active segments (FOV) could be modified. For example, a central LIDAR segment n=1 to the left and n=1 to the right defined three active LIDAR segments, which defined a joint FOV = 12°. However, imposing medium and large obstacles on the workspace affected the LIDAR measurements heavily. Still, the size of the obstacles was not the only relevant factor. The distance from the LIDAR to the obstacle also played a crucial role, as the further away the obstacle was, the less the FOV was affected. Therefore, the distance from the LIDAR to the obstacle and its size were relevant factors in realizing the influence of the obstacles. Practical experiments have shown that, whether just a single or two LIDAR FOV segments were affected, no additional error was caused due to the obstacles. Therefore, the human feet and overturned chair as obstacles imposed far away from the mobile robot, e.g., more than 3 m, did not impose any additional errors. On the other hand, the human feet and the chair imposed on the LIDAR at a distance of 0.5 m spoiled the measurement completely.

#### 3.3.6. Comparison Between the Odometrical Calculations and PSO Calculation of the Mobile Robot Positions and Orientations

Finally, a comparison was carried out of the calculated positions and orientations between the robot’s built-in odometrical algorithm as a benchmark and PSO-ALM. The experiment was performed as follows: The robot was reset at the pose M1. Then, it was controlled manually to the next M-pose consecutively and stopped there. The robot underwent all M-poses. The OA was used to calculate the position and orientation sequentially at each pose when stopped. The error was calculated for each pose for both methods and these are collated within Table 4. $\left|\Delta x\right|$, $\left|\Delta y\right|$, and $\left|\Delta \omega \right|$ and their averages are shown for all M-poses. Further discussion is provided in the Discussion Section.

Direct comparison between the odometrical algorithm and the PSO-ALM showed that the odometrical errors increased rapidly after the recalibration, i.e., the initialization at pose M1. In absolute terms, their average values were more than twice as high as for the PSO-ALM. In general, the longer the path, the larger the odometrical algorithm error due to disturbance integration and more frequent wheel slippage. As the PSO-ALM localization is absolute, the error remained stable across the whole path. From the table, one can notice that, in any case, when moving a robot incrementally from a single point to another point, the maximum distance error does not exceed 15 cm, nor does the rotation error exceed 15°, therefore defining the sufficient error margins.

#### 3.3.7. Comparison of the PSO-ALM Against the Plain Vanilla PSO, GA, GEO, GA-ALM, and GEO-ALM

Similar nature-inspired OAs were used as benchmark algorithms for our PSO-ALM algorithm. The benchmark algorithms used for the tests were as follows: the plain vanilla PSO, the vanilla plain GA with added elitism and adaptive mutation rate, the plain vanilla GEO and its combination with the ALM extension to prevent trapping in the local minima: PSO-ALM, GA-ALM and GEO-ALM. We also compared the odometrical built-in algorithm along with the previously mentioned six OAs to see their suitability for the indoor localization application on the mobile robot Pepper. We checked the accuracy of the measured position M5 (see Figure 14), how many times on average the algorithms were trapped in the local minima in the 10 real lab tests per checked OA, performed as described in the Section 3.3.3, and the minimum and maximum overall elapsed times for the code written in the Python language. We observed only the measured position M5 due to our experiences that this was the position where the OAs were often trapped in the local minima between all seven measured positions. The time of execution for the measurement of all the LIDAR measurements for FOV = 44° and Wi-Fi data transfer (IEEE 802.11 a/b/g/n, Security: 64/128 bit: WEP, WPA/WPA2) to the PC computer (Intel(R) Core(TM) i7-14700, 2.53 GHz, RAM 31.7 GB, Windows 11), where the OAs were executed, lasted app. 0.18 s. This was the same for all the algorithms, except the odometrical algorithm, which was executed on the built-in computer of the mobile robot Pepper and did not need to be executed on the remote PC. Table 5 shows the comparison data.

## 4. Discussion

We have proposed a novel PSO-ALM to cope with the absolute localization of a mobile robot with low-quality LIDAR measurements. The experimental work was divided into simulations and real lab experiments. The simulation tests modeled both random perturbations of the LIDAR as well as camera mapping workspace corner definitions. The former was to model the random disturbances caused by the LIDAR inaccuracies. The latter was used for workspace mapping—localization itself was not capable of simultaneous mapping; therefore separate mapping of the workspace (also called corner identification) was conducted before. The real laboratory tests included manual identification of the workspace corners, as well as automated identification using 3D camera mapping.

The simulation tests have shown that random perturbations of $pc_{L}=\pm 1\%$ of simulated LIDAR measurements have quite a small effect on the deterioration in accuracy of the calculated OA positions and orientations. Increasing the perturbations $pc_{L}$ lowered the quality of the results slightly but did not prevent the adequate localization skills of the robot. Even in the case of the highest perturbation, $pc_{L}=\pm 10\%$, the deviations were less than or equal to 8 cm; i.e., only a single case had $\left|\Delta x,\;y\right|\leq 8$ cm and $\left|\Delta \omega \right|=5.2{}^{\circ}$. Definition of the workspace corners had a dominant effect on the localization accuracy.

Increasing the perturbation of the corners of the workspace in simulations from $pc_{c}=\pm 1\%$ to $pc_{c}=\pm 5\%$ or $pc_{c}=\pm 10\%$ caused a significant deterioration in the accuracy of the calculated PSO-ALM. Simulated deviations of up to $\left|\Delta x\right|=23$ cm and $\left|\Delta \omega \right|=5.5{}^{\circ}$ were spotted in the worst-case scenario. For the practical lab experiments, these accuracies of identifying the corner positions using the ORB-SLAM2 were estimated to be $pc_{c}=\pm 15\%$, even larger than the simulated ones. The estimated errors in the positions and orientations reached $\left|\Delta x\right|=39$ cm, $\left|\Delta x\right|=23$, cm and $\left|\Delta \omega \right|=8.7{}^{\circ}$ for orientation in the worst case. Still, the general shape of the workspace was recognizable, and the OA was functioning, but the quality of the solutions was worse than expected. An adequate definition of the robot’s workspace area must be of higher priority than the LIDAR measurements; hence, significant care must be taken in the proper definition of the robot’s workspace area.

The real lab tests were much affected by the heavily deteriorated LIDAR measurements, especially for distances greater than 2 m and angles of measurement greater than 44°. An experiment was performed to realize the adequate FOV, where the disturbances were lowest. Here, the quality function value $Q_{e}$ was implemented to measure the disturbances. A separate experiment to realize the adequate PSO-ALM parameter settings was performed too. The influence of the obstacles imposed in front of the LIDAR were also studied carefully. The closer the obstacle, the greater the disturbance and the worse the localization inaccuracy. Despite the inaccurate LIDAR measurements, the PSO calculations of the mobile robot positions and orientations were still close to the ground-truth measurements of the real mobile robot positions and orientations (see Figure 14). The average absolute errors in the base coordinate system were ($\left|\Delta x,\;\Delta y\right|=3.43$) cm for all seven measurements, and the average absolute error was ($\left|\Delta \omega \right|=2.92{}^{\circ}$).

The value of elapsed time $t_{e}$ was decreased by lowering the FOV settings, as expected. The most adequate FOV setting seemed to be FOV=44° for lab measurements as a compromise between time complexity and sensitivity to obstacles. The FOV setting could be decreased further to FOV=20°, decreasing the time elapsed for measurements $t_{e}$ significantly and increasing the quality function value $Q_{e}$ of the measurements due to less dispersed beams but increasing the sensitivity to obstacles. By using more accurate LIDAR sensors, the proposed algorithm would have produced fewer local minima solutions and converged toward the global minimum faster. Therefore, we can say that the proposed method could be generalized, as it should work even better with more accurate LIDARs.

The experiments performed have shown that the best quality function value for pose P1 $Q_{e-P1}$ was obtained by a population size NP=50 or NP=70 and number of iterations $N_{i}=100$. Increasing the population size further did not improve the result; actually it worsened the time complexity. Although it was not mentioned in this paper, the real lab experiments were solved using the traditional genetic algorithm (GA) for a fair comparison. The GA performed similarly in terms of the quality function value $Q_{e}$, but with significantly higher overall elapsed time $t_{o}$, app. 3 to 4 times larger than the PSO.

Extensive testing was performed for pose P1 with multiple local optima within the workspace. Additionally, convergence plots were generated for the fitness function value for position P1. Figure 12 represents the two possible outputs, with one of them being the local optimum trapped solution and the other the global optimum. The experiments performed for position P1 show the fitness function value for both experiments and the positions and orientations calculated by the plain vanilla PSO for each value of the fitness function. Executing the PSO twice, and monitoring the performance after 160 iterations, these fitness function values were between ff=0.132 and ff=0.136 for both experiments, with the difference that one of these was the local optimum trapped solution and the other was the global optimum solution. The wrong positions and orientations were calculated four times more often than the correct one due to the local optimum solution having a fitness function value lower than the global optimum solution. This was a significant drawback of the plain vanilla PSO algorithm. Hence, the integrity check and PSO-ALM were introduced to verify the correctness of the calculated results, as by referencing the fitness function value only, it was impossible to deduce whether the solution was trapped or not. The PSO-ALM demonstrated superior performance compared to the odometric method and overperformed all the benchmarks, the GA, GEO, GA-ALM, and GEO-ALM.

## 5. Conclusions

The absolute localization of the semi-humanoid mobile robot Pepper in the reference Cartesian coordinate system was presented in this paper. Three sets of light detection and ranging (LIDAR) distance measurements were used, i.e., a single measurement ahead of the robot and two measurements to the sides of the robot. Underlying simulations were performed, as well as real laboratory experiments. Based on the knowledge obtained from the simulations, the modified particle swarm optimization algorithm with a local optima avoidance mechanism (PSO-ALM) and integrity check for verifying the correctness of the solution was proposed and tested in a real-world robot workspace. The proposed solution was compared to the plain vanilla PSO, which underwent local optima trapping. Experimentally, we realized that the solution trapped into the local optima was more than four times more frequent than the global optimum. The built-in odometrical algorithm, on the other hand, depended much more on the distance traveled, since it underwent the classic disturbance integration that increased with respect to the distance. The PSO-ALM worked well when knowing the workspace with high accuracy. When a low-accuracy workspace plan, obtained from the automated ORB simultaneous localization and mapping, was given to the PSO-ALM, its performance decreased heavily. This is one of the drawbacks of the PSO-ALM that should be addressed in future, i.e., to allow for both simultaneous localization and mapping, not only the localization itself. Although some cues were written in this paper, a rigorous statistical analysis with time complexity of the proposed algorithm still needs to be performed. Another improvement that could be implemented is the fusion of localization data coming from different sources for improved reliability, e.g., using an extended Kalman filter or similar. Data fusion of the odometry with the LIDAR seems a natural choice for a future work. The odometry is accurate enough for short distances but prone to drift—such drift could be reset occasionally using the LIDAR localizations. If successful, the proposed method should be validated in a three-dimensional workspace in the future, including the *z* axis for localization of airborne unmanned aerial vehicles.

## Figures and Tables

**Table 1:** OAs’ parameter settings. The parameters were determined based on extensive experience with the aforementioned algorithms, complemented by the experimental methods.

Parameter	Descriptor	Description	Value
Population Size	NP	Number of particles/individuals	70
Max Iterations/Generations	$N_{i}$	Maximum number of iterations	100
Dimension	*D*	Number of dimensions (x,y,ω)	3
Inertia Weight	$c_{0}^{\left(PSO\right)}$	Weight of inertia	0.8
Cognitive const.	$c_{1}^{\left(PSO\right)}$	Weight for personal best	2.1
Social const.	$c_{2}^{\left(PSO\right)}$	Weight for global best	1.1
Neighborhood const.	$c_{3}^{\left(PSO\right)}$	Weight for neighborhood best	0.8
Initial mutation rate	$p_{m}^{\left(GA\right)}$	Initial mutation rate for GA	0.001
Adaptive mutation factor	$F_{m}^{\left(GA\right)}$	Adaptive mutation factor for GA	2
Crossover rate	$p_{c}^{\left(GA\right)}$	Probability of crossover	1
Elitism	$E^{\left(GA\right)}$	Number of elite individuals	1
Propensity to cruise	$p_{c}^{\left(GEO\right)}$	Initial and final propensities	0.5, 0.5
Propensity to attack	$p_{a}^{\left(GEO\right)}$	Initial and final propensities	2, 2

**Table 2:** The influence of $N_{i}$ and NP on $Q_{e-P1}$. Remark: The value av. repeated was measured as the average value between N=11 repeated values, which all always gave the same $\left|\Delta x\right|$, $\left|\Delta y\right|$, and $\left|\Delta \omega \right|$, which presents the high repetitiveness of the PSO-ALM.

NP	$N_{i}$	|Δx| [cm]	|Δy| [cm]	|Δω| [°]	$\bar{\mathrm{repeated}}$	$Q_{e-P1}$
70	20	0	2	4.0	2.05	2.01
	40	4	0	1.5	2.30	1.95
	60	3	1	2.1	2.85	2.23
	80	4	1	1.9	2.45	2.33
	100	3	1	1.9	1.29	1.79
	120	4	1	1.9	1.95	2.21
	140	4	1	1.9	2.35	2.31
	160	4	1	1.9	2.10	2.25
50	20	3	1	1.4	2.31	1.93
	40	9	6	7.2	3.78	6.49
	60	3	2	1.3	2.05	2.08
	80	3	1	2.0	3.41	1.85
	100	3	0	1.8	1.95	1.69
	120	4	1	1.9	2.85	2.43
	140	4	1	1.8	2.80	2.40
	160	4	1	1.9	1.65	2.14
30	20	7	2	6.7	4.55	5.06
	40	1	1	3.8	3.15	2.23
	60	3	3	1.3	3.15	2.68
	80	4	1	2.0	3.05	2.26
	100	4	1	1.9	3.30	2.55
	120	4	1	1.9	2.50	2.60
	140	4	1	1.9	2.00	2.23
	160	4	1	1.9	2.50	2.35

**Table 3:** Time complexities and quality function values $Q_{e}$ with respect to the scanning angle FOV (sample size N=11). The average elapsed time $\bar{t_{e}}$ and average overall time $\bar{t_{o}}$ were measured from the MATLAB 2023a environment. Note that the similar execution of the FOV = 44° scenario in Python took significantly less time, $\bar{t_{o}}=0.46$ s (see the Supplementary Material Video S1).

Scanning Angle (FOV) [°]	$\bar{t_{e}}$ [s]	$\bar{\mathrm{repeated}}$	$Q_{e}$	$\bar{t_{o}}$ [s]
60	1.12	1.28	3.69	2.55
44	0.98	1.71	2.88	2.66
28	0.78	1.71	3.12	2.11
20	0.61	2.48	2.95	2.12
12	0.54	1.00	6.31	1.08

**Table 4:** Comparison between the odometrical algorithm and the PSO-ALM.

Position	Odometrical Algorithm			PSO-ALM		
	|Δx|[cm]	|Δy|[cm]	|Δω|[°]	|Δx| [cm]	|Δy| [cm]	|Δω| [°]
M1	0	0	0	3	2	2.8
M2	1	4	8.3	4	3	3.4
M3	8	4	10.0	9	1	2.2
M4	13	14	9.7	3	1	4.2
M5	9	13	9.2	1	1	0.4
M6	4	2	9.2	3	8	3.7
M7	17	10	18.4	1	8	3.7
**Average**	7.42	7.83	6.7	3.43	3.43	2.92

**Table 5:** LM count = Local Minima count, i.e., number of times the OA was caught into the local optima, $t_{o}$ = overall elapsed time in seconds.

OA	LM Count [%]	Best Δx [cm]	Best Δy [cm]	Best Δω [°]	$t_{o}\left[s\right]$
PSO	60.8	1	1	0.4	0.2–0.3
GA	48.2	2	1	1.2	0.6–1.2
GEO	80.0	4	4	1.7	0.4–0.6
PSO-ALM	0	1	1	0.4	0.5–1
GA-ALM	0	2	1	1.2	1.8–4.8
GEO-ALM	0	4	4	1.7	1.3–2.6

## Data Availability

The original contributions presented in this study are included in the article/Supplementary Material. Further inquiries can be directed to the corresponding author.

## Supplementary Materials

The following supporting information can be downloaded at https://www.mdpi.com/article/10.3390/s25206283/s1, Video S1: Demo of the mobile robot Pepper going through the M-poses within the workspace when performing the localization process.

## Abbreviations

The following abbreviations are used in this manuscript:

PSO	Particle swarm optimization
PSO-ALM	PSO with local minima avoidance mechanism
GA	Genetic algorithm
GA-ALM	GA with local minima avoidance mechanism
GEO	Golden eagle optimization

GEO-ALM	Golden eagle optimization with local minima avoidance mechanism
M-poses	Stationary poses within the workspace for mobile robot localization
LIDAR	Light detection and ranging
SLAM	Simultaneous localization and mapping
V-SLAM	Visual SLAM
RANSAC	Random sample consensus
MCL	Monte Carlo localization
FOV	Field of view
ff	Fitness function
$Q_{e}$	Quality function value
$Q_{e-P1}$	Quality function value for pose P1
